# Critical Remarks on the "Plea of Insanity"

**Published:** 1855-07-01

**Authors:** Richard Poole

**Affiliations:** Fellow of the Royal College of Physicians, Edinburgh, formerly Superintendent of the Royal Lunatic Asylum, Montrose.


### CRITICAL REMARKS ON THE "PLEA OF INSANITY."

BY RICHARD POOLE, M.D.,

*Fellow of the Royal College of Physicians, Edinburgh, formerly Superintendent of the Royal Lunatic Asylum, Montrose.*

(*Continued from page* 281.)

More recent in date (1831) is that of George Waters, reckoned "similar" by Alison, who thus relates it. "The pannel, in a rude and strange manner, had taken his son by the hand, who was playing near the Water of Leith, at Bonnington, and had a fork in his possession. As he got nearer the place where the fatal deed was perpetrated, his look appeared, to the witnesses who saw him, wilder and more frantic. He was seen looking into the water, where the body was thrown after it was committed, in a raised and insane manner. He was seen near the spot, in a subsequent part of the same day, moving about like a deranged person, and declared he was Sir William Wallace, and an honour to his country. When apprehended, he admitted having killed his son, made no resistance, spoke incoherently, and prayed aloud. The evening before he had spoke very insanely about having been at Inchkeith on a raft. In November, 1829, he had been committed for disturbing his neighbours; he had then the appearance of *delirium tremens*, and was confined in a strait-waistcoat; and his relations had subsequently written to the Leith Police to look after the pannel, as he could not take care of himself. In these circumstances, the insanity was clearly proved, and so the jury held, with the approbation of the court; and he was confined for life!" The circumstances, beyond all doubt, demonstrated insanity, as understood by medical men, but, by no means, absolute alienation of reason, sought for by some lawyers. The former, we shall immediately see, may err, like the latter, in regard to the existence of the disease. "It is by no means unusual," says Alison in continuance, "to find instances of persons committing crime under the influence of insanity, who yet give no indication of it when conversing in jail with a medical man." Every one will be prepared for such a remark who understands the well-known fact that the malady, though ever so intense, admits of intervals or remissions of manifestation, and, generally speaking, requires peculiar excitement in order to display itself unequivocally. Alison gives an example of the kind, in the case of Janet M'Callum, September, 1829.

"She was charged with having stolen a child belonging to her mistress, as well as a quantity of clothes from her house. Insanity was pleaded in bar of trial; but, after the examination of a single witness, who said he saw nothing insane about her, it was withdrawn, and she pleaded guilty. The case, however, was certified to the high court (from the circuit at Stirling, it appears), to give time to investigate the state of the woman's mind, which was very suspicious from the style of her declaration, and from most of the stolen articles having been found torn to pieces in the wood near her master's house. It ultimately turned out that she had been insane, and escaped some months before from a lunatic asylum near Greenock, and had been considered insane all her life. Still, the medical men who examined her in Edinburgh declared they did not regard her as 'void of reason;' but, as the crime had evidently been committed in a state of mental alienation, the prosecutor did not move for sentence, and she was confined till her sanity was restored." The case was given on Sheriff Alison's own report, and, probably, is one in which he was counsel. I could wish for more information respecting it, and, especially, touching the grounds on which the medical men in Edinburgh declared the woman not "void of reason." The same thing, I may remark, might be predicated of nearly all the patients (amounting to hundreds) who ever came under my care, though, most assuredly, insane and certified to be so by competent members of the medical profession; and, I verily believe, a like testimony will be given by the majority—possibly all—of those individuals who have held, or now hold, superintendence in lunatic asylums. Of course, idiotic and purely demented persons must be excluded from the list.

"Somewhat of the same description," according to Alison, "was the case of James Cummings, 12th January, 1810, charged with murder" (reported, I presume, in a subsequent edition of Hume's work). Some years before, it seems, he "had met with a severe injury on the head, but had recovered, enlisted, and was not considered by his fellow-soldiers as insane; but he was silent, solitary, and quarrelsome when in liquor. One morning, when on guard as a sentinel, being teased by a fellow-soldier, he became suddenly outrageous, and pursued him into the barracks. Having arrived there, he pushed at a woman with his bayonet, and missed her, but immediately after struck at a fellow-soldier coming out of a door, and killed him on the spot. The jury, by a plurality of voices, found the pannel insane at the time of committing the murder; but there seems good ground for Baron Hume's opinion, that it would have been more agreeable to law to have found him guilty, but recommended him, on account of a constitutional irritability, arising from his wound, over which he had no control, to the Royal mercy." More agreeable to law—very possibly, I would say; but neither to common sense nor to justice, if, as was admitted in the preceding case, "the crime had evidently been committed in a state of mental alienation." Surely, "a constitutional irritability, arising from a wound, over which (meaning, I take for granted the former) the man had no control," was as valid a plea as the condition of the woman against whom "the prosecutor did not move for sentence. I confess myself unable to discover any fixed, not to say, rational, rubric, by which both Hume and Alison are guided in deciding on such matters. But to proceed.

"Insanity was clearly proved," says the latter, "in the case of William Douglas, May 28, 1827, who had set fire to the furniture of the lodgings which he occupied at Peebles, and nearly burned the house. He was convicted of attempt at fire-raising, but, in consequence of his state of mind, ordered to be confined for life." This needs no comment.

"The law of England," we are now told by Sheriff Alison, "is founded on the same principles;" and he gives us, in illustration, the well known case of Hadfield, indicted for shooting at the king in Drury-Lane Theatre (1800). The details are unnecessary here. "It was quite clear," says Alison, "that this man was mad, and his case was eloquently pleaded by Lord Erskine. Lord Kenyon held that, as the prisoner was insane immediately before the offence was committed, it was probable that he had not recovered his senses at the time he fired, and that, as there was no reason to believe that he had recovered his sanity in the interval between the two events, he was entitled to an acquittal, which he accordingly received, and was ordered to be confined for life." But how, I ask, does this tally with the opinion of Baron Hume, above approved, in favour of a verdict of guilty, accompanied by a recommendation to the Royal mercy? Farther, too, I would ask, if, in his conduct throughout, Hadfield did not exhibit a good deal of method and design—evidently the product of reason—therefore showing its existence, though, undoubtedly, struggling against or labouring under delusion?

Hitherto we have had under review the first general rule, or position laid down by Alison—that, namely, which insists on insanity being "of such a kind as entirely deprived the person of the use of reason as applied to the act in question, and the knowledge that he was doing wrong in committing it," in order to "a complete bar to punishment." We have now to consider the second, announced in these words, "If it appear from the evidence that the pannel, though partially deranged, was not so much so as to relieve him entirely from punishment, the proper course is to find him guilty; but, on account of the period of infirmity of mind, which he could not control, recommend him to the royal mercy"—a suggestion, it will be observed, very similar to what has already been given on the authority of Baron Hume.

We are told, in exposition, that "cases frequently occur in the highest degree perplexing both to the court and jury, which can only be justly resolved by an application of the principle and mode of proceeding above set forth. They are those in which the accused was to a great degree to blame, but would not probably have committed the fatal act but for some constitutional or supervening derangement which rendered him not so *far responsible* (thus marked in *italics* by Alison) as those who, by enjoying their reason unclouded, have no defence whatever against atrocious actions. In such cases there is a mixture of guilt and misfortune; for the former he should be severely punished, for the latter the extreme penalty of the law should be remitted. This can only be effected by adopting the course above pointed out." It will be speedily seen, however, that this course has not always been successful in issue, and, indeed, one might have anticipated a difficulty in reconciling severe punishment, supposing it just, with a remission of penalty. Possibly, however, Alison means only *condemnation* on one hand, and *pardon* on the other. If so, the certainty of the alliance, it would seem, cannot be reckoned on: and, consequently, juries may become of opinion that, when they really desire to show mercy, they must provide for it by their verdict. Can they ever hesitate to do so in the face of evidence which satisfies them of the existence of such infirmity of mind as could not be controlled? How shall they draw a line of distinction between that state and the condition of one who, though understanding right and wrong, is yet unable to use reason in a special act? And will not a "constitutional or supervening derangement," sufficient to exempt from some responsibility, because, in its absence, the accused would probably not have committed a certain deed, appear to be a very good ground for suspecting that the whole evil depended on it, and, therefore, that no punishment should follow? Of course, some exceptions must be made, as, possibly, in a few of the cases to which I now hasten. "Thus, in the case of William Gates, 21st Dec., 1811, who was tried for shooting his wife with a musket, insanity was pleaded in bar of trial, but failed. On the evidence, it appeared that whisky and consequent irritability of temper, had a large share in the deed, but that, even when sober, he was of a melancholic temperament, and not like other men. The jury found that the act was committed in a state of insanity. But Baron Hume's opinion is obviously well-founded, that they should have convicted and recommended to the royal mercy." But why so, one might reasonably ask, if they were satisfied by evidence of insanity at the time, and that, even when sober, the pannel "was not like other men"?

"In like manner," continues Alison, "in the case of Pierce Hoskins, 23rd April, 1812, who was tried for the murder of his own child, of four years old, in a fit of drunken insanity, it appeared that the pannel, when intoxicated, was perfectly mad for days together, and in that state he committed the fatal deed. He was acquitted by the jury; but Baron Hume declares that it is questionable whether an assize do right when they sustain the plea of this lower degree of infirmity of mind, exasperated only into a short fit of outrage and fury by excess of liquor; or where they receive as evidence the atrocity or brutality of the act itself that has been done, though there have been no previous symptom of the disease." A lower degree of infirmity of mind! Why, it appears, he was "*perfectly mad* for days together, and *in that* state committed the fatal deed." In the absence of details, probably well known to Hume, I can say nothing on the last point in his remarks.

"The latter course," says Alison, meaning what Hume advised, "was followed in the case of Alexander Campbell, 18th December, 1809, who was found guilty of robbery, but recommended to mercy, 'on account of a certain degree of weakness of intellect, to which he appears to be subject,' and received, in consequence, a transportation-pardon"—no doubt, it may be imagined, better than hanging, as in days of yore, but still a severe punishment, and, one would think, very unsuitable for a man of weak intellect.

"In like manner, in the case of Susan Tilly, 11th March, 1816, a more rational verdict was returned. It there appeared, from the testimony of two physicians and a surgeon, who had visited the pannel in jail, that she was of a weak mind, laboured under religious dreams, spoke of her interviews with the devil, said he had tempted her to burn the barn, and that God had reproved her by scorching her hands on the occasion. On other subjects, however, she reasoned correctly, and knew the distinction between right and wrong. She was convicted, but recommended to mercy, and received in consequence a pardon from the Crown." So far well and happily, I would say; but her manifest insanity, as judged by medical men, would have warranted a different verdict, and secured what *might not* have been granted; for though, as Alison says, "the same course was followed at Jedburgh, autumn, 1831, in the case of Samuel Rogers, he was not quite so fortunate. "He was accused of murdering an Irish reaper, in the course of harvest, whom he pursued into the river Tweed; and a considerable degree of insanity was proved at the trial. The jury found the pannel guilty, 'but, in respect of the *alleged* insanity, recommend him to mercy.'" We shall be somewhat enlightened here by Alison, who himself reports the case. "This way of wording the verdict was incorrect; but their meaning evidently was that a *certain degree* of insanity only was proved, insufficient to liberate the pannel from punishment altogether, but sufficient to excuse him from the extreme penalty of the law." It may be so; but they had better have expressed themselves by leaving out a word liable to misinterpretation; and, still more, in my humble opinion, by an acquittal, if satisfied as to "a considerable degree of insanity." My reason appears in what follows:— "The case was not so viewed in the proper quarter, for he was executed in pursuance of his sentence"—guilt, doubtless, being deemed to preponderate over misfortune. But, pray, even admitting the excess, was not something due to the less weighty element, according to the course approved? Perhaps—but we are not told so—the poor man had the comfort of a silken halter!

Sheriff Alison generalizes from such examples in relation to a special point thus—"This seems the proper way of resolving those cases, unhappily too numerous, in which a fatal act has been committed in the course of a temporary fit of insanity, arising from excessive drinking. In all such cases there is room for a distinction. If the pannel, naturally sane, has been rendered mad *solely by drink*, and this infirmity was known to him, he seems to have no defence whatever against the legal punishment of his actions; for it is the duty of every man to abstain from indulgences which lead to perilous consequences; and as intoxication is no defence, so the insanity consequent upon its excessive and criminal indulgence seems to be as little. But, on the other hand, if either the insanity has supervened from drinking, without the pannel's having been aware that such an indulgence, in his case, leads to such a consequence, or if it has arisen from the combination of drinking with a half crazy or infirm state of mind, or a previous wound, or illness, which rendered spirits fatal to his intellect, to a degree unusual in other men, or which could not have been anticipated, it seems inhuman to visit him with the extreme punishment which was suitable in the other case. In such a case, the proper course is to convict, but, in consideration of the degree of infirmity proved, recommend to the royal mercy."

I reckon it unnecessary to dwell on these various considerations—liberal, generally speaking, as they are—farther than to say that, while *wilful drunkenness* is unquestionably immoral in itself, and perhaps, therefore, with propriety deemed by the law rather an aggravation than an alleviation of a criminal charge, as Alison afterwards mentions, the habit of drinking to excess is, in many instances, the consequence of or an attendant on real mental disorder, arising from other and very different circumstances. The whole subject, in truth, is beset with difficulties, to which I can only point in this most superficial manner.

Alison's third general proposition is in these terms:—"If the pannel, though somewhat deranged, is yet able to distinguish right from wrong, in his own case, and to know he was doing wrong in the act which he committed, he is liable to the full punishment of his criminal acts." This is nearly to the same purport as that of a former statement, or may be deduced from it; and, accordingly, says our author, "It has been already noticed that the true test of insanity is to be found, not in the ability to distinguish between right and wrong in the general case, but with reference to the particular case of the pannel; and that he is amenable to the same punishment as other men, when his conscience tells him, or is in a situation to have told him, that what he did was wrong. But anything short of this complete alienation of reason will be no defence; and mere oddity of manner, or half craziness of disposition, if unaccompanied by such an obscuring of the conscience, will not avail the prisoner. This is proved by a multitude of cases, both in the Scottish and English practice." Simply remarking, what might be shown by analysis, that Alison does not here express himself throughout with perfect accuracy, I go on to the cases considered in point. They are those of Thomas Gray and Robert Bonthron (for which see my notes on Hume), then we have that of Sir Archibald Kinloch, introduced by the explanatory observation, "It is not indispensable that the madness should be continued in respect of time, so as it be clearly established at the date of the crime." Following Hume again, Alison tells us that "the plea of insanity must be received with much more difficulty in cases proceeding from the desire of gain, as theft, swindling, or forgery, and which generally require some art and skill for their completion, and argue a sense of the advantage of acquiring other people's property"—details being added of the cases of Thomas Henderson (as in Hume), John Smith, spring, 1827, and Alexander Duff, spring, 1829, which latter two claim attention. They are preceded by a remark to the effect that "such a defence, as was made in the former, has been very frequently attempted in subsequent cases, but hardly ever with success," for a reason stated—"it is difficult to figure that state of mental alienation which leads pannels to lay their hands on other people's property, or, if they labour under such an illusion as made them mistake it for their own, which induces them to adopt the art, skill, and concealment necessary for its effectual perpetration. Such cases, however, do sometimes occur," as, for example, in Smith, charged with horse-stealing, but evidently insane, and treated as such; then, as to Duff, similarly charged, having stolen a horse out of a stable in the night, "and, with some art, having untied the door, which was fastened with a string, but he had afterwards abandoned it on the roadside, where it was found next morning among some corn, at the distance of five miles from the place of theft." "The whole circumstances," continues Alison, "evinced a disordered mind, and the charge, in consequence, was not insisted on by the prosecutor;" adding, as a general principle, "In all cases where such a defence is pleaded, the great thing to attend to is the subsequent conduct of the pannel, and whether he evinced any symptoms of conscious guilt, or a desire to conceal what had been done subsequent to its commission; for, if he did, it is difficult to see how the plea can be well-founded, that he knew not the criminal nature of his actions." I shall offer only two short remarks on the whole of this deliverance, for, against such authority, "established, moreover," as Alison notices, "in the English practice," it would be vain to argue. The first is, that no one accustomed to see maniacs can have the least difficulty in figuring to himself the very state of mental alienation referred to as a sort of improbability, more especially if he take into account, as he ought and will, the existence of various propensities—moving powers—whatever they may be, and however denominated, totally distinct from reason or judgment. And, secondly, I have to say, as also matter of experience, that "the great thing," on which Alison relies as conclusive, is in truth quite fallacious, worthy of no confidence in determining the sanity of an individual at the time of committing any deed, however criminal and atrocious. In other words, subsequent conduct, to the amount of entire rationality, is perfectly compatible with previous derangement; and, in point of fact, which Alison seems to have overlooked when making one of the above statements, some of the cases recorded give no small support to the position now maintained. I shall allude to one only, because what he says of it is peculiarly cogent in the matter. It is that of Sir A. Kinloch, in which the jury found insanity proved, "though he regained his senses completely a short time after the melancholy event."

Among the English instances decided on the same principles which have ruled in Scotland, we have, first, that of Lord Ferrers, tried for murder before the House of Peers. "It was proved that he was occasionally insane, and incapable of knowing what he was doing (one might have expected this to be held sufficient excuse); but the murder was deliberate, and, when he committed the crime, he had capacity sufficient to form a design and know its consequences. He was found guilty, and executed."

2. Arnold, charged with shooting at Lord Onslow. "It clearly appeared that the prisoner was, to a certain extent, deranged, and that he had greatly misconceived Lord Onslow's conduct, but formed a regular design, and prepared the proper means for carrying it into effect. He was convicted, but, at Lord Onslow's intercession, reprieved, and confined for life." In this case, Mr. Justice Tracy laid it down to the jury, "that the defence of insanity pleaded against a great offence must be clearly established; that it is not every idle and frantic humour of a man which will exempt him from being accountable for his actions, but such a deprivation of reason as renders him as an infant, a brute, or a wild beast, incapable of knowing what he was doing—a condition, I unhesitatingly affirm, such as is not exemplified in one out of a hundred persons requiring and actually receiving humane treatment, with the kindliest sympathy, in our large asylums for lunatics. At the time of writing this sentence, ninety-four patients were under my own care; a few of them—three or four—were altogether or almost entirely fatuous; but, even comprehending these—because still indicating a portion of intellect—I might have safely said that none realized the character of an irresponsible maniac, such as Mr. Justice Tracy describes.

3. Parker, indicted for entering the service of France, then at war with this country. His defence was insanity. He had been weak from infancy, and it had been thought surprising that he was received into the army. But he had deliberately entered the foreign services, and knew what he was doing, stating as a reason, that it was "more agreeable to be at liberty and have plenty of money, than be at want in a dungeon." He was convicted, "under the direction of the court, that insanity was not established." Alison makes no comment on the case. I will do so, but it shall be short. The man's reasoning was precisely that of a madman; indeed, quite like the process adopted by a clergyman, recorded by Dr. Abercrombie, and to which I may advert hereafter.

4. "Bowler's case, 2nd July, 1812, accused of shooting Mr. Burrowes, was one of considerable difficulty," according to Alison. "Insanity, occasioned by epilepsy, was the defence pleaded. He had an epileptic fit in July, 1811, and since that time had been very strange in his demeanour, eating his meat almost raw, and lying on the grass exposed to the rain, and so dejected that it was necessary to watch him lest he should destroy himself." All this, it might be hoped, would have been reckoned potent enough. But there was more. "A commission of lunacy was produced, dated 17th June, 1812, on which the prisoner was found insane from 30th March last. Mr. Warburton, the keeper of a lunatic asylum, had no doubt of the insanity of the prisoner, and stated that persons subject to that species of madness often took strong antipathies, founded on illusions totally destitute of foundation." Not a doubt of it—Mr. W. was quite correct. But notwithstanding, "the jury, after much deliberation, found the prisoner guilty." In this case, it seems, "Mr. Justice Le Blanc laid it down to the jury, that they had to determine whether the prisoner, when he committed the offence, was incapable of distinguishing right from wrong, or under the influence of an *illusion*, in respect to the prosecutor, which rendered his mind at the moment insensible to the nature of the act he was about to commit, since in that case he would not be legally responsible for his actions; but that, if he was not under such an illusion, or not incapable of understanding the distinction between right and wrong, he was amenable to punishment." Alison adds—"This appears the true view of the subject." One would like to know how the "much deliberation" of the jury depended on this charge.

5. The noted case of Bellingham, who shot Mr. Percival in 1812. "Insanity was pleaded to the jury, and many strong facts brought out in support of the plea, tending to show that the prisoner falsely imagined himself subject to a long series of injuries from that minister." His fate is well known. In his case, Lord Chief-Justice Mansfield laid it down to the jury, that, "in cases of murder, it must be proved beyond all doubt that the prisoner, at the time of committing the act, did not consider that murder was a sin by the laws of God and nature; that lunatics, as long as they can distinguish right from wrong, are answerable for their conduct; and that the mere fancying of a series of injuries which did not exist, was no defence against the charge of murder, if the prisoner were in other respects capable of distinguishing right from wrong." Let us see what Sheriff Alison says on these sentiments, with which, almost evidently, he is not quite satisfied. "On this case it may be observed, that unquestionably the mere fancying a series of injuries to have been received will not serve as an excuse for murder, for this plain reason, that, supposing it true that such injuries had been received, they would have furnished no excuse for the shedding of blood; but, on the other hand, such an illusion as deprives the pannel of the sense that *what he did was wrong* amounts to legal insanity, though he was perfectly aware that murder in general was a crime; and therefore the law appears to have been more correctly laid down in the cases of Hadfield and Bowles than in this instance, though no injustice may have been committed in the actual result." No injustice may have been committed in the actual result—simply—a hanging!—though the verdict was decidedly influenced by a legal opinion, not "the most correct," seeing there was a better, and though many strong facts sustained the plea! Alas—alas! I shall, probably, either find or take occasion to show how indignantly, and yet how justly, at the distance of several years, Lord Brougham expressed himself respecting the deplorable trial of Bellingham. Sheriff Alison here closes the English cases, and his third main proposition. In relation to one point connected with the former examples adduced, I have a special reason, which may afterwards be patent, for quoting the opinions of an author whose judgment in such matters is worthy of most serious regard.

"The subject of hallucination, in insanity, may be either entirely imaginary and groundless, or may be a real event viewed in false relations and carried to false consequences. This view of the subject bears upon an important point which has been much agitated—the liability of maniacs to punishment—and which has been ably and ingeniously argued by Lord Erskine in his defence of Hadfield. The principle contended for by this eminent person is, that when a maniac commits a crime under the influence of an impression which is entirely visionary and purely the hallucinations of insanity, he is not the object of punishment; but that, though he may have shown insanity in other things, he is liable to punishment, if the impression under which he acted was true, and the human passion arising out of it was directed to its proper object. He illustrates this principle by contrasting the case of Hadfield with that of Lord Ferrers. Hadfield had taken a fancy that the end of the world was at hand, and that the death of his Majesty was in some way connected with important events which were about to take place. Lord Ferrers, after showing various indications of insanity, murdered a man against whom he was known to harbour deep-rooted resentment, on account of real transactions in which that individual had rendered himself obnoxious to him. The former, therefore, is considered as an example of the pure hallucinations of insanity; the latter as one of human passion founded on real events, and directed to its proper object. Hadfield, accordingly, was acquitted, but Lord Ferrers was convicted of murder and executed. The contrast between the two cases is sufficiently striking; but it may be questioned whether it will bear all that Lord Erskine has founded upon it. There can be no doubt of the first of his propositions, that a person acting under the pure hallucinations of insanity, in regard to impressions which are entirely unfounded, is not the object of punishment (meaning ought not to be so). But the converse does not seem to follow—namely, that the man becomes an object of punishment merely because the impression was founded on fact, and because there was a human passion directed to its proper object. For it is among the characters of insanity, not only to call up impressions which are entirely visionary, but also to d stort and exaggerate those which are true, and to carry them to consequences which they do not warrant in the estimation of a sound mind. A person, for instance, who has suffered a loss in business, which does not affect his circumstances in any important degree, may imagine, under the influence of hallucination, that he is a ruined man, and that his family is reduced to beggary. Now, were a wealthy man, under the influence of such hallucination, to commit an outrage on a person who had defrauded him of a trifling sum, the case would afford the character mentioned by Lord Erskine—human passion founded upon real events, and directed to its proper object; but no one, probably, would doubt for a moment that the process was as much the result of insanity as if the impression had been entirely visionary. In this hypothetical case, indeed, the injury, though real, is slight; but it does not appear that the principle is necessarily affected by the injury being great, or more in relation to the result which it leads to according to the usual course of human passion. It would appear probable, therefore, that, in deciding a doubtful case, a jury ought to be guided, not merely by the circumstances of the case itself, but by the evidence of insanity in other things. This, accordingly, appears to have been the rule on which a jury acted in another important case mentioned by Lord Erskine, in which an unfortunate female, under the influence of insanity, murdered a man who had seduced and deserted her. Here was a real injury of the highest description, and human passion founded upon it and directed to its proper object; but the jury, on proof of derangement in other things, acquitted the prisoner, who accordingly soon passed into a state of 'undoubted and deplorable insanity.' In the case of Lord Ferrers, also, it would appear that the decision proceeded, not so much upon the principle of human passion directed to its proper object, as upon an impression that his lordship's previous conduct had been indicative of uncontrolled violence of temper rather than actual insanity."—(*Dr. Abercrombie, Intell. Powers.*)

The remaining propositions set forth by Sheriff Alison scarcely come in my way. But they may be quoted with the briefest possible annotations.

"(4.) The proof of insanity it lies upon the pannel to establish; and, in the case of an insane person having lucid intervals, it lies upon him to show that the criminal act was committed during the continuance of the disease, unless the intervals were of short duration. On part of which—or generally—he adduces Hume's remark as appearing well founded—namely, in reference to the pannel being bound to substantiate his defence if the lucid intervals were long, whereas the reverse is the case where they are extremely short, 'and he was apprehended shortly after the act in a state of furiosity,' thus, namely, 'that the point should be left for the consideration of the jury, rather than made the subject of unbending presumptions which must, in many instances be unsuitable to the justice of the particular case with which they are intrusted." The *extension* of this remark, I am disposed to think, would be equally proper, to say the least.

"(5.) Insanity may be pleaded in bar of trial, if the pannel be then insane, and the Court, *ex proprio motu*, will take cognisance of the state of a prisoner's mind, if he appear incapable of conducting his defence." Here it is observed, as comparatively a recent thing, that "proof (of insanity) may competently be brought forward by any one capable of speaking to the point, whether contained in the list of witnesses or not; and the proof is taken by the Court itself, without the intervention of an assize." This was first adopted in 1801, and has been since followed.

"(6.) Where the trial goes on, and insanity is found proven by the jury, the Court orders the prisoner to be confined for life, or until caution is found by his friends to put him in a place of safe custody during the remainder of his life." On this point I need not speak. As to sundry and important topics connected with, or proceeding from it, everybody knows many volumes have been, and everybody will expect to be, written. My present object keeps me aloof. It being understood, and Sheriff Alison having stated that, on matters regarding the plea of insanity, there is a correspondence or essential agreement between the laws, as well as the practice, of Scottish and English Courts, I append to these remarks an extract from the *Times* of 20th June, 1843, setting forth minutes of proceedings in the House of Lords, when the Judges delivered their replies to certain questions on the subject.

"The House of Lords met yesterday morning at 11 o'clock, to hear the opinions of the Judges on several questions relating to crimes committed by persons supposed to be insane, or afflicted with monomania. There was a full attendance of peers, amongst whom were Lord Brougham, Lord Cottenham, Lord Melbourne, Lord Campbell, Lord Wynford, Lord Kenyon, and others.

"His Majesty the King of Hanover (who came down to the House exactly at 11 o'clock on horseback, attended by two grooms in undress liveries) was also present, and sat on the woolsack by the side of the Lord Chancellor. His Majesty paid the most marked attention to the reading of the opinion of the Judges by the Lord Chief Justice Tindal.

"His Royal Highness the Duke of Cambridge was also present.

"Mr. Justice Maule, at some length, but in a low tone of voice, stated his reasons for differing with his learned brethren on the questions which had been submitted to their consideration. His Lordship said, that with reference to the fifth and last question proposed—viz., Can a medical man, conversant with the disease of insanity, who never saw the prisoner previously to the trial, but who was present during the whole trial and the examination of all the witnesses, be asked his opinion as to the state of the prisoner's mind at the time of the commission of the alleged crime, or his opinion whether the prisoner was conscious at the time of doing the act that he was acting contrary to law? or whether he was labouring under any, and what, delusion at the time?—he had no hesitation in saying that such a question could legally be put to a witness. It had been the practice to adopt that course. He had no knowledge of such questions having been successfully objected to. The fact of the Lord Chief Justice of the Court of Common Pleas, and the other distinguished Judges who presided with him on the trial of M'Naughten, having allowed such questions to be put (to Dr. Forbes Winslow), was to his mind a sufficient proof of their legality.

"Lord Chief Justice Tindal then rose and said, that Her Majesty's Judges had most carefully and attentively considered the questions which had been submitted to them by their Lordships respecting insane persons accused of crimes. and, with the exception of his learned brother, Mr. Justice Maule, they were unanimous in the opinion which he was then instructed to read to the House. It was not necessary on that occasion to enter into the facts of any particular case; it would be wrong to do so, as there was such an endless variety, all and each attended with such improbable and different circumstances, that no general rule could be laid down. Every case must be decided by its own particular circumstances. His Lordship said, as the subject was about to come under the consideration of Parliament, the Judges had not lost any time in considering the questions submitted to them: and as they were unanimous, with the exception, as he before said, of Mr. Justice Maule, they did not consider it necessary to give their opinions *seriatim*. The first question propounded for their consideration was as follows:—

"'What is the law respecting alleged crimes committed by persons afflicted with insane delusion in respect of one or more particular subjects or persons: as, for instance, where at the time of the commission of the alleged crime, the accused knew he was acting contrary to law, but did the act complained of with a view, under the influence of insane delusion, of redressing or revenging some supposed grievance or injury, or of producing some supposed public benefit?'

"With regard to this question the opinion of the Judges was, that notwithstanding the party committing a wrong act when labouring under the idea of redressing a supposed grievance or injury, or under the impression of obtaining some public or private benefit, he was liable to punishment.

"Second question—'What are the proper questions to be submitted to the jury, when a person alleged to be afflicted with insane delusion respecting one or more particular subjects or persons is charged with the commission of a crime, murder for example, and insanity is set up as a defence?'

"The Judges, in answer to this question, wished him to state that they were of opinion that the jury ought in all cases to be told, that every man should be considered of sane mind, unless it was clearly proved in evidence to the contrary. That before a plea of insanity should be allowed, undoubted evidence ought to be adduced that the accused was of diseased mind, and that at the time he committed the act he was not conscious of right or wrong. This opinion related to every case in which a party was charged with an illegal act, and a plea of insanity was set up. Every person was supposed to know what the law was, and therefore nothing could justify a wrong act, except it was clearly proved the party did not know right from wrong. If that was not satisfactorily proved, the accused was liable to punishment, and it was the duty of the Judges so to tell the jury when summing up the evidence, accompanied with those remarks and observations as the nature and peculiarities of each case might suggest and require.

"With regard to the third question—viz., 'In what terms ought the question to be left to the jury, as to the prisoner's state of mind at the time when the act was committed?'—the Judges did not give an opinion.

"The fourth question was—

"If a person under an insane delusion as to existing facts, commits an offence in consequence thereof, is he thereby excused?'

"The answer to this question was, that the Judges were unanimous in opinion that, if the delusion was only partial, that the party accused was equally liable with a person of sane mind. If the accused killed another in self-defence, he would be entitled to an acquittal, but if committed for any supposed injury, he would then be liable to the punishment awarded by the laws to his crime.

"With regard to the last question—

"'Can a medical man, conversant with the disease of insanity, who never saw the prisoner previously to the trial, but who was present during the whole trial and the examination of all the witnesses, be asked his opinion as to the state of the prisoner's mind at the time of the commission of the alleged crime, or his opinion whether the prisoner was conscious at the time of doing the act that he was acting contrary to law, or whether he was labouring under any, and what, delusion at the time?'

"The Judges were of opinion that the question could not be put to the witness in the precise form stated above, for by doing so they would be assuming that the facts had been proved. That was a question which ought to go to the jury exclusively. When the facts were proved and admitted, then the question, as one of science, could be generally put to a witness under the circumstances stated in the interrogatory.

"Lord Brougham said the House and the country were under great obligations to the learned Judges for the care and attention they had given to the subject, and therefore moved that the opinions read by the Lord Chief Justice be entered on the journals, as he was certain that an almost unanimous opinion would be found of the greatest advantage when in future legislating on the subject.

"Lord Campbell was glad this momentous question had been submitted to the consideration of the Judges. They had been asked their opinion as to the existing law, and the answer. to him, was most satisfactory. They were not requested to give any opinion as to future legislation.

"Lord Cottenham, Lord Wynford, and the Lord Chancellor, expressed similar opinions.

"The opinion of the Judges was then ordered to be printed and entered on the journals."

The questions, it seems then, were five in number; and on four of these, there was unanimity of opinion in the interpretation of the law by the Judges. The sole difference, however, is a very important one, because relating to the actual application of the law in courts, Mr. Justice Maule, the dissentient, plainly referring to a practice which the other Judges represent as illegal. The third question, be it said somewhat paradoxically, was not answered at all. The first, touching responsibility, in a manner involving the whole, is consequently of highest value—yet, singularly enough, the reply to it does not introduce the clause which relates to the special ground at issue, namely, "the influence of insane delusion," which is conspicuous in two places of the query. Thus —"What is the law respecting alleged crimes committed by persons afflicted with *insane delusion*," &c. &c. and "Did the act complained of with a view, under *the influence of insane delusion*," &c. &c. Answer—"Notwithstanding the party committing a wrong act when labouring," &c. &c. (See again the series at large.)

Now, besides the omission, which leaves the answer in the state of a mere truism, if taken without reference to the question itself, the collocation of words seems to me peculiarly ill-chosen. For what, strictly construed, do they really mean? In fact, that the party committing a wrong act is liable to punishment —a position disputed by no one, and, therefore, not here required. The intended meaning, on the contrary, clearly is that, notwithstanding the party labouring under the idea of redressing, &c. &c. when committing a wrong act, he was liable, &c.; or, as might have been expressed, "the (for a) party committing (or who committed) a wrong act, when labouring under the idea, &c. is nevertheless liable," &c. But criticism of this kind—and there might be more —though surely fair where public interests are so much concerned, is vain; for faulty as the language commented on may be, no one can absolutely mistake what the Judges meant—namely, that if, at the time of committing the alleged crime, the accused knew he was acting contrary to law, he is liable to punishment, even although he then laboured and acted under the influence of an insane delusion. And accordingly the answer to the second question expressly says, "that before a plea of insanity should be allowed, undoubted evidence ought to be adduced that the accused was of diseased mind, and that, at the time he committed the act, he was not conscious of right or wrong." "Nothing could justify a wrong act, except it was clearly proved the party did not know right from wrong. If that was not satisfactorily proved, the accused was liable to punishment."

But farther, and conclusively as to a large class of cases, to the 4th question, "If a person under an insane delusion," &c.—the reply is such as to leave no room for doubting—"If the delusion was only partial," &c.; to which is added, almost unnecessarily, one would imagine, "If the accused killed another," &c. The 5th answer strikes me as being somewhat ambiguous, or, rather, not in strict connexion with, or appropriate to, the question, which relates to the medical opinion itself; whereas, the Judges, in the first place, say that "the question *could not be put* to the witness in the precise form stated;" and then, that "when the facts were proved and admitted, then the question, as one of science, *could be generally put* to a witness, under the circumstances," &c. Now, what are these? The medical man is said to be "present during the whole trial and the examination of all the witnesses;" while, what is asked of him —not, be it observed, in any precise form—is simply "his opinion as to the state of the prisoner's mind at the time," &c. or, "whether the prisoner was conscious at the time," &c. or, whether he was labouring under any, and what delusion at the time. But, according to the supposition of the question, the medical man must have had the facts before him (he having been present, as above stated), or, in other words, the facts are supposed to be proved and admitted. When, then, do the Judges mean he is to be asked his opinion? Observe the very question, which is not to be put in the precise form, &c. In short, no small explanation is needed in the whole affair—more light, with greater distinctness of language; and I, for my own part, though willing to concur with Lords Brougham and Campbell, in saying that the House and the country were under great obligations to the Judges for the care and attention they had given to the subject, cannot honestly congratulate them on account of clear and satisfactory results.*

* It is proper to mention that there are different versions of the opinions—a circumstance in itself unhappy, and calculated to bewilder the public mind, already distracted enough on this highly painful topic. In order to mitigate, or, rather, entirely arrest the censure which might visit daring opposition to the decrees of certain eminent legal authorities, I avail myself of some of the sentiments uttered, on a remarkable occasion, by the Hon. Thomas (afterwards Lord) Erskine. They are, in themselves, exceedingly cogent. He is alluding to Lord Chief Justice Hale, who held, that prisoners should be acquitted only when a total and permanent want of reason was proved; and to Mr. Justice Tracey, according to whom, a man, to be exempted from penal consequences, must be one that is totally deprived of his understanding and memory, and does not know what he is doing any more than an infant, than a brute, or a wild beast! Now, how did the eloquent and justly-successful advocate meet these sad dogmas? "If a total deprivation of memory (he of course comprehends 'understanding') was intended by these great lawyers to be taken in the literal sense of the word: if it was meant that to protect a man from punishment, he must be in such a state of prostrated intellect as not to know his name, nor his condition, nor his relation towards others; that, if a husband, he should not know he was married, or, if a father, could not remember he had children; nor know the road to his house, or his property in it,—then no such madness ever existed in this world."

Again. "In all the cases which have filled Westminster Hall with the most complicated considerations, the lunatics and the other insane persons who have been the subject of them, have not only had memory, in my sense of the expression;

